# Hospital Professionals as Dual Agents: A Superordinate Identity to Solve Interprofessional Conflicts in Hospitals? Comment on "Dual Agency in Hospitals: What Strategies Do Managers and Physicians Apply to Reconcile Dilemmas Between Clinical and Economic Considerations?"

**DOI:** 10.34172/ijhpm.2022.6928

**Published:** 2022-03-02

**Authors:** Rosalía Cascón-Pereira

**Affiliations:** Business Management Department, University Rovira i Virgili, Reus, Spain.

**Keywords:** Dual Agent, Professional Hybrid, Identity, Healthcare Management, Collaborative Models, Interprofessional Conflict

## Abstract

The inherent conflict between economic and clinical considerations, between professionalism and managerialism, and between being a manager or being a clinician is widely acknowledged in the sociology of professions. The original article by Waitzberg and colleagues focused on how hospital professionals reconcile these conflicting demands. In this commentary, we argue that their assumption that the considered hospital professionals (managers, chief financial officers [CFOs], chief physicians and practising physicians) are dual agents moves on from the unproductive debates of inherent conflicts to envisage possibilities of reconciling economic and clinical considerations. We conclude that the instrumental use of the term dual agent to include "the other" (the manager or the clinician) in a superlative and inclusive category can be considered a reframing strategy to solve inherent interprofessional conflicts and to implement more collaborative models in healthcare.

## Introduction

 In their recent article entitled “Dual Agency in Hospitals: What Strategies do Managers and Physicians Apply to Reconcile Dilemmas Between Clinical and Economic Considerations?” Waitzberg and her colleagues^1^ discuss how some hospital professionals (managers, chief financial officers [CFOs], chief physicians and practising physicians) mitigate dilemmas by reconciling economic and clinical considerations in their daily decision-making in the context of activity-based payment in German and Israeli hospitals. As the article recognises, the inherent conflict between clinical and economic issues in healthcare management research has been extensively covered by the extant literature.^2,3^ However, how these conflicting demands can be reconciled and integrated is still a pending research issue as the article correctly points out. By addressing this pending issue, the article makes a practical and deliberate contribution to the field of healthcare management. This commentary first reflects on why this issue has remained unexplored for so long by referring to the literature on the sociology of professions that has focused on the inherent conflicts between professionalism and managerialism, clinical and economic considerations, physicians and managers. Second, it brings to the fore an unintended contribution of the article: the effects of applying the term “dual agent”, traditionally used in applied health economics, to refer to all hospital professionals regardless of their professions. In particular, we point out that this assumption may be a reframing strategy so that professional hybrids are included in a more inclusive and superordinate category with other hospital professionals. Although unintentionally, this re-labelling of professional hybrids as dual agents might help alleviate conflicts between traditional professional identities such as clinician and manager and to solve conflicts between economic and clinical issues by integrating both objectives. Indeed, the article by Waitzeberg et al may pave the way, perhaps serendipitously, towards collaborative healthcare models that focus on interprofessional cooperation rather than on conflict.

## Doctor Managers as Professional Hybrids in the Literature on the Sociology of Professions

 In the literature on the sociology of professions, medical doctors or physicians are conceived as professionals. “Professional” has traditionally been considered as an exclusive identity developed through qualifications, training and socialization, creating social identity boundaries.^3,4^ Doctor managers, those physicians with managerial responsibilities, have been considered in this literature as professional hybrids across different health systems the world over – the Netherlands,^5^ the United Kingdom,^2,6^ the United States,^7^ New Zealand^8^ and Spain^9^ – and referred to by a wide range of different titles: physician executives (the United States), medical managers, doctor managers, medical directors, clinical directors, chief physicians or ward directors. Hybrid roles are framed by both professional and managerial logics in healthcare systems.^3^ One of the hot topics in the literature has been to explore the conflicting demands placed on them by economic and clinical issues, their conflicting identities as clinicians and managers, the interprofessional conflict with senior managers and the inherent conflict between managerialism and professionalism.

 Identities are the meanings that individuals attach reflexively to their selves as they seek to answer questions such as “Who am I?” “Who are we?”^10^ Professional identities are therefore associated with the enactment of professional roles.^11^ Conflicting professional identities have been identified in the case of hybrid roles such as doctor managers. Do they define themselves as doctors or as managers?^9^ A wealth of studies have explored the consequences of this inherent conflict in their attitudes towards management, in their relationship with hospital senior managers and even in hospital’ performance.^2,3,5-9^

 Hospitals are professional organizations and as such are very bureaucratic although decision making is decentralized. They are complex organizations because they are made up of different healthcare professional groups (physicians, nurses, managers, pharmacists, social workers, physiotherapists, psychologists, etc) with power and autonomy, and these groups have diverging agendas, values, perspectives and goals that make conflict inherent in their relationships. So, hospitals are complex scenarios where different professional identities are at play and frequently in conflict.

 Overall then, the focus in the literature on the sociology of professions and organization management has been on conflict rather than on HOW to solve the main inherent conflicts, on uniprofessional identities rather than on more superlative interprofessional identities.^15^ In this scenario, the article we discuss here opens up new ways of eliminating inherent conflicts by considering professional hybrids as “dual agents.” In the section below, we reflect on how the commented article does this, maybe unintentionally but with powerful effects and future prospects for healthcare management strategic interventions and collaborative models.

## Doctor Managers as Dual Agents Like Other Hospital Professionals: A Reframing Strategy to Solve Inherent Interprofessional Conflicts in Hospitals?

 The commented article reframes the extant conflicting scenario by referring to doctor managers as dual agents, a term typically applied to physicians in health economics and health policy^13^ to recognise the conflicting principal-agent problems of third-party payment and information asymmetry of the physician, who is at the same time an agent for the patient but also an agent for insurance companies. By definition, the concept of dual agent is broader than the concept of professional hybrid so it can be seen as a superordinate category.

 What is extremely interesting in the commented article is that by assuming that all hospital professionals (managers, CFOs, chief physicians and practising physicians) are dual agents because they are committed to both patients and the hospitals where they are employed,^1^ it also implicitly assumes that managers, physicians and professional hybrids equally try to reconcile patients’ clinical needs and the quality/safety of care with economic considerations. In so doing, they are unintentionally applying a powerful conflict management strategy which is the dual identity model of conflict resolution,^14^ used in most cyst-shaped political conflicts around the globe. According to this model, conflict is seen to revolve around counterposed social identities defined at subgroup (in our case, physician or manager) and superordinate levels. And the key to satisfactory conflict resolution lies not in increasing the salience of a single superordinate social identity (that of dual agent who shares the same problems with other hospital professionals) at the expense of a subgroup identity (clinician or manager), but in acknowledging and expressing both superordinate and subgroup identities. This is the key to effective interprofessional teams in healthcare. Indeed, there needs to be a salient superordinate identity so that parties can share a common motivation to reconstruct social differences as sources of strength rather than bones of contention, and group differences can be recognised (different clinical and managerial expertises) as part of the shared superordinate social identity. By recognising that they are clinicians or managers but also dual agents who have to reconcile patients’ clinical needs with economic considerations, the commented article unintentionally applies a reframing strategy for conflict resolution among different professional groups in the hospital context. The social identities of clinicians and managers are harnessed towards a common goal: the alignment of clinical and economic considerations to attain hospital goals. By reframing the way hospital professionals are seen (as dual agents and not as managers, clinicians or professional hybrids), Waitzberg and her colleagues focus on their commonalities rather than on their differences as different professional groups with different objectives. Hence, they deconstruct the dichotomy of two opposing professional groups (clinicians and managers) and help solve interprofessional conflicts. Thus, the choice of the term “dual agent” to refer to all hospital professionals, if made on purpose, can be seen as a reframing strategy to align the objectives of these professional groups and bring them together. It is also a good example of the instrumental use of language to include “the other” (the manager or the clinician) in the superlative and inclusive category of dual agent. This strategy can also be applied to other healthcare professionals (nurses, psychologists, physiotherapists, social workers, etc) who face the same conflicting dilemmas between economic and clinical considerations.

 It is also interesting to notice that this original assumption that all hospital professionals are dual agents has led the authors to focus on different issues and get different outcomes from studies usually undertaken within the sociology of professions and organization management. In this literature, clinicians and managers are usually conceived as opposing professional groups with different and conflicting objectives, and chief physicians or doctor managers are conceived as professional hybrids. In particular, they formulate two research questions: (1) In which situations are economic and clinical considerations aligned and in which situations do dilemmas exist between economic and clinical considerations? (2) What strategies do hospital professionals use to balance these considerations in their daily decision making? Underlying these questions, there is the assumption that since they are all dual agents there is the possibility of reconciling economic and clinical considerations. Should the starting point for this research and the underlying assumption have been different, the focus of the research and therefore the results would also have been different. From the paradigm of social constructionism, this leads us to reflect on the assumptions we make when doing research and the implications these assumptions have.

 Moreover, the commented article is a good illustration of cross-fertilization between disciplines. In particular, it shows how a term originating from one discipline (Applied Health Economics) can be applied to problems in another (Sociology of Professions).

 Finally, we hope this commentary has contributed to highlighting the potential impact of the Waitzberg et al article not only on advancing understanding of the possibilities of reconciling economic and clinical considerations but also on designing strategic interventions in healthcare policy and management to reduce conflict between different healthcare actors. In this regard, this commentary may be of interest for healthcare managers and policy-makers in their attempts to bring the efforts of all healthcare actors together. Assuming that all hospital professionals share the same difficulties and dilemmas, and creating a superordinate identity for them as dual agents opens up a fertile line of healthcare management and policy interventions. The strategic choice of a name to refer to all hospital professionals can elicit the salience of different social identities and, consequently, different organisational dynamics, which will lead to more collaborative healthcare models.^15^ This strategy together with the interprofessional socialization of physicians at individual, professional and system levels ^4^ can foster the needed changes for the 21st century health service delivery to move from the established discourse of professionalism and conflict to a culture of interprofessional collaboration. However, more needs to be done with evidence-based studies to inform policy-makers and healthcare managers of the practical utility of reframing professional hybrids as dual agents who have to rise to the same hospital challenges and difficulties, and thus have to work together to do so. Although the commented article has unintentionally opened up possibilities for strategic interventions to reduce inherent conflicts in hospitals, it is important for researchers and policy-makers to understand the social identity dynamics elicited by choosing a name to refer to all hospital actors.

## Ethical issues

 Not applicable.

## Competing interests

 Author declares that she has no competing interests.

## Author’s contributions

 RCP is the single author of the paper.
